# Traditional Chinese Herb Combined with Surgery versus Surgery for Varicocele Infertility: A Systematic Review and Meta-Analysis

**DOI:** 10.1155/2015/689056

**Published:** 2015-01-29

**Authors:** Rong-liang Dun, Min Yao, Long Yang, Xue-jun Cui, Jian-min Mao, Yu Peng, Guang-chong Qi

**Affiliations:** ^1^Department of Urology, Yueyang Hospital of Integrated Traditional Chinese and Western Medicine, Shanghai University of Traditional Chinese Medicine, Shanghai 200032, China; ^2^Spine Disease Institution, Longhua Hospital, Shanghai University of Traditional Chinese Medicine, Shanghai 200032, China

## Abstract

*Objective*. The objective of this study was to conduct a systematic review to assess the effectiveness and safety of traditional Chinese herb combined with surgery for male varicocele infertility compared to surgery. *Methods*. Randomized controlled trials (RCTs) data of traditional Chinese herbs combined with surgery for male varicocele fertility versus surgery were collected by searching the Cochrane Library, Embase, PubMed, and Chinese databases. The risk of bias was assessed using Cochrane Handbook. Study outcomes were presented as risk ratios (RRs) for dichotomous data. *Results*. Seventeen of 72 potentially relevant trials met the inclusion criteria. The methodological qualities of the RCTs were low. Compared with the surgery group, the traditional Chinese herb combined with surgery group had superiority in pregnancy rate at 3-month (RR = 1.76, and *P* = 0.008), 6-month (RR = 1.58, and *P* = 0.0005), and 2-year (RR = 1.58, and *P* = 0.0005) follow-ups. No RCT was found to describe the side effects. *Conclusion*. On considering the low methodological quality of RCTs, there was no enough evidence on traditional Chinese herb with surgery for male varicocele infertility, and more high-quality RCTs of large sample sizes are required.

## 1. Introduction

Varicocele, abnormally dilated scrotal veins, is the most prevalent abnormal physical finding in male infertility. Fifteen percent of the normal male population is accounting to have varicocele and approximately 40 percent of the men present with infertility [1, 2]. Infertility affects 10–15% of couples endeavoring to conceive, with male infertility contributing to nearly 50% [3]. However, the exact pathophysiology of varicocele-induced damage is not yet completely understood. Varicocele seems to cause testicular damage through multiple simultaneous mechanisms, and all of which may result in male infertility. Hence, it is unsurprising that varicocele has been considered a potential cause of infertility in male patients [3].

Treating male factor infertility should is an ultimate goal to achieve a live birth. There are two approaches to repair varicocele: surgery and percutaneous embolization [4]. Surgical treatment is the gold standard; however, controversy still remains regarding the benefit of varicocele repair to improve the male fertility [5]. According to the results of numerous reports and systematic reviews, varicocele repair has not shown to improve the conception rate [6–9]. After surgical treatment, about 50% of the patients failed to achieve fertility, and hence surgery is not an ideal treatment to improve infertility [6, 9]. All types of varicocele surgery are associated with a small risk of wound infection, hydrocele, persistence or recurrence of varicocele and, rarely, testicular atrophy [10]. As pathogenesis of varicocele causing infertility is not clearly understood and there is an irregular association between the degree of varicocele and sperm quality of infertility patients, surgical indication has not been considered a unique standard [5].

Some infertile couples may turn to Eastern medical approaches such as traditional Chinese herb, an integral part of Traditional Chinese Medicine (TCM), in an attempt to become pregnant, as these treatments may be perceived as being lower cost, safer, or more effective [11–14]. Currently, the physiological mechanisms for most traditional Chinese herbs used in male infertility are unknown. Substantial pharmacological and chemical researches have been performed to investigate the chemical constituents of a variety of traditional Chinese herbs. Many of the traditional Chinese herbs have strong antioxidant activity [15] and function like gonadotropins, which can increase the production of testosterone and improve the sperm motility.

In recent years, a large number of clinical reports of Chinese herb combined with surgery in the treatment of varicocele infertility were published; however, its efficacy- and safety-related systematic evaluation was poorly stated. This systematic review aimed to evaluate the effectiveness and safety of traditional Chinese herb combined with surgery for male varicocele infertility compared to surgical treatment.

## 2. Materials and Methods

### 2.1. Sources

The following electronic databases were searched from the beginning date of the databases till July 15, 2014: PubMed, Embase, the Cochrane Central Register of Controlled Trials, China National Knowledge Infrastructure (including China Doctoral/Master Dissertation Full-text Database and China Proceedings of Conference Full-text Database), VIP Journal Integration Platform, Wanfang Data, and Chinese Biomedical databases. The text terms including varicocele, infertility, male, traditional Chinese herb, and randomized controlled trials were used to identify the relevant information. There were no restrictions on time or language. The clinicaltrials.gov web was also searched for unpublished completed trials. The references of all selected publications and reviews were manually searched for further relevant articles. Publication languages and types (including conference proceedings, abstract only articles, or theses) were not limited as long as they met the following inclusion criteria.

Two independent reviewers (Rong-liang Dun and Min Yao) performed the literature review of the titles and abstracts of potentially relevant references to determine whether they met the eligibility criteria for inclusion.

### 2.2. Study Selection

#### 2.2.1. Types of Studies

Randomized controlled trials (RCTs) were included. Quasi-RCTs, non-RCTs, or randomized trials with false randomization methods were excluded.

#### 2.2.2. Types of Participants

Male patients of child-bearing-age couples with a marital life for more than 2 years but with no pregnancy or the couples with a history of pregnancy but with no more pregnancy in the last 2 years would be considered as infertile. Adult male patients diagnosed with varicocele infertility based on any set of explicit criteria were included, while other infertilities such as immune infertility and other unexplained infertilities were excluded.

#### 2.2.3. Types of Interventions

Treatment groups received both traditional Chinese herbs and surgery. The dosage, formulation, route of administration of traditional Chinese herbs, and the type of surgery were not been limited.

#### 2.2.4. Types of Control Treatment

Any type of surgery for varicocele infertility without other treatments was considered as control group.

#### 2.2.5. Types of Outcome Measures

Fertility rate at 3 months (short-term), 6 months (medium-term), and 2 years (long-term) after follow-up and the side effects of interventions were evaluated.

Studies were excluded if they (1) did not provide information concerning evaluation rates or pregnancy; (2) did not include experimental treatment intervention using traditional Chinese herb combined with a surgery and surgery alone as control; and (3) were animal trials.

### 2.3. Data Extraction

All data were independently abstracted by two investigators (Long Yang and Jian-min Mao) using a predefined data extraction form. Disagreement was resolved by discussion or consensus with a third reviewer (Guang-chong Qi). Data including first author's name, publication year, sample size, patient characteristics, components of Chinese herbal medicine, and measured outcomes were extracted. Missing information was sought by contacting the corresponding authors of the studies.

### 2.4. Risk of Bias Assessment

Risk of bias assessment was independently performed by two independent reviewers (Rong-liang Dun and Min Yao) and disagreements were solved by consensus or by a third reviewer (Guang-chong Qi). The Cochrane Handbook for Systematic Reviews of Interventions was used to assess the risk of bias of each trial [16].

Seven domains were assessed as follows.

1. Was the allocation sequence adequately generated?
2. Was the allocation adequately concealed?
3. Were the patients and personnel blind from allocation adequately?
4. Was the outcome assessment blind from allocation adequately?
5. Were incomplete outcome data adequately addressed?
6. Were reports of the study free of suggestion of selective outcome reporting?
7. Was the study free of other problems that could put it at a risk of bias?

The answer “yes” indicated a low risk of bias (Y), “unclear” indicated that the risk of bias was uncertain (U), and “no” indicated a high risk of bias (N).

### 2.5. Data Analyses

For meta-analysis, pregnancy rates of dichotomous data (e.g., pregnancy rate at 3 months after intervention) were pooled using risk ratio (RR). All statistical analyses were performed using Review Manager 5.2.1 software (Cochrane Collaboration, Oxford, UK).

The final results were compared to assess the differences between the intervention and control groups. The Cochrane's *χ* ^2^ test and *I* ^2^ were used to assess heterogeneity. If the *P* value was less than 0.10 or if the *I* ^2^ value was above 50%, there was a considerable level of heterogeneity, respectively, for *χ* ^2^ test and *I* ^2^ test [16]. Clinical heterogeneity was assessed by reviewing the differences in the distribution of participants' characteristics among trials (age, gender, specific diagnosis/diagnostic subtypes, and duration of disorder and associated diseases). Clinically and statistically homogeneous studies were pooled using the fixed effects model, while clinically and statistically heterogeneous studies were pooled using the random effects model.

### 2.6. Publication Bias Assessment

Funnel plots were generated if sufficient trials (at least 10) were available to assess the publication bias and other related biases.

## 3. Results

### 3.1. Study Selection

**Figure 1:**
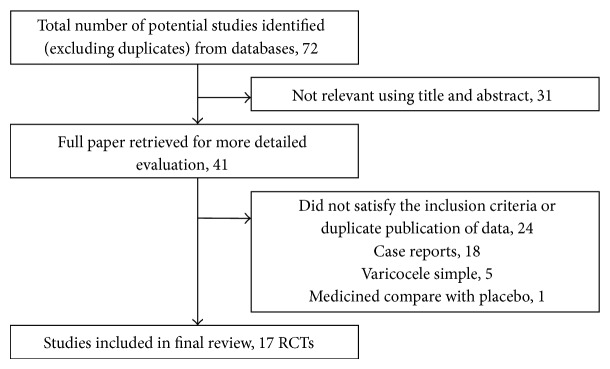
Summarization of the process of literature identification and selection.

From a total of 56 titles, 41 studies were deemed to be potentially relevant and the full-text articles were read to confirm their eligibility. Among 41 studies, 24 studies were excluded, including 18 case reports, 5 studies with patients having simple varicocele, and 1 study comparing treatment intervention with placebo. Finally, 17 trials met the inclusion criteria (Figure 1).

### 3.2. Study Characteristics

All of these 17 trials were published in Chinese language. A total of 1541 participants were included in these trials, with 794 in traditional Chinese herb combined with surgery group and 747 in surgery group. The sample sizes of these trials ranged from 40 to 225. There were 8 trials reporting that the female factor was excluded through gynecological examination [17–24], while the remaining studies reported that the female fertility factors were normal with no more description.

The components of traditional Chinese herbs used in each trial were different. The most common form of traditional Chinese herbs used in 8 trials was decoction, like Bushenquyushengjin decoction [25], Shengjingzhuyu decoction [26], and Tongpizanyu decoction [17]. Other forms of traditional Chinese herbs used in clinical trials were granules in two trials [27, 28], capsules in three trials [29–31], pills in four trials [18, 31–33], tablets in one trial [19], and injection in one trial [25] (Table 1).

### 3.3. Risk of Bias Assessment

**Figure 2:**
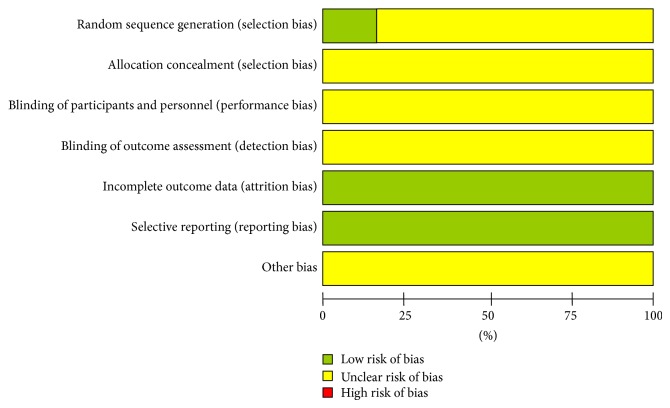
Risk of bias graph.

The methodological quality of the trials varied stable (Figure 2), which ranged from 3 to 4 low risk of bias. Methodological quality of all included trials was poor as no study was of high quality.

Although all included trials reported randomization, only three of them had adequately described the randomization method, with computer-generated random numbers [21, 27, 32]. Moreover, none of studies had reported information including allocation concealment, blinding of participants and study personnel, and blinding of outcome assessment. All of the relevant trials had adequately addressed the incomplete outcome data and it was the same with selective reporting.

There were no other biases found in these trials; however, considering the poor methodological quality of the trials, unclear risk of bias was given to all included trials.

### 3.4. Primary Outcomes

#### 3.4.1. Pregnancy Rate at 3-Month Follow-Up

**Figure 3:**
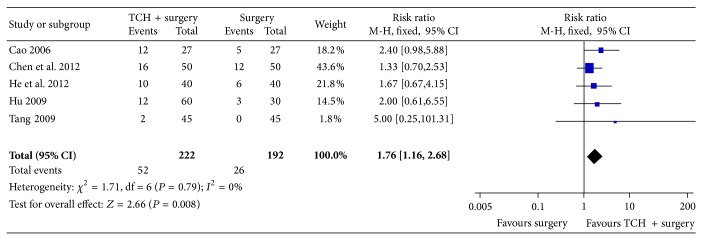
Meta-analysis of pregnancy rate at 3-month follow-up.

For the clinical pregnancy rate at 3-month follow-up, data were available from 5 trials with 414 patients. Pooling of the results from these trials showed no significant difference in the clinical pregnancy rate at 3-month follow-up between the traditional Chinese herb combined with surgery and surgery alone groups (RR = 1.76, 95% CI = 1.16 to 2.68, and *P* = 0.008; Figure 3) using the fixed effects model.

#### 3.4.2. Pregnancy Rate at 6-Month Follow-Up

**Figure 4:**
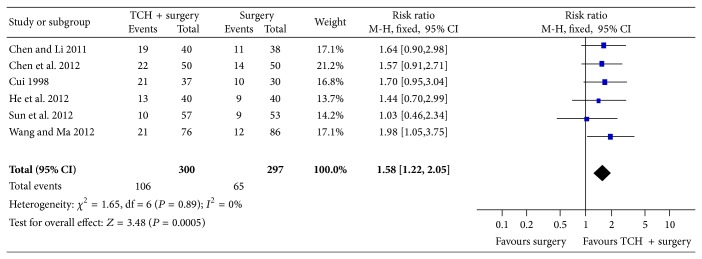
Meta-analysis of pregnancy rate at 6-month follow-up.

There were six RCTs with 597 patients that reported pregnancy rate at 6-month follow-up. When the effects of traditional Chinese herbs combined with surgery versus surgery on pregnancy rate at 6-month follow-up using the fixed effects model were compared, the meta-analysis revealed significant differences (RR = 1.58, 95% CI = 1.22 to 2.05, and *P* = 0.0005; Figure 4).

#### 3.4.3. Pregnancy Rate at 2-Year Follow-Up

**Figure 5:**
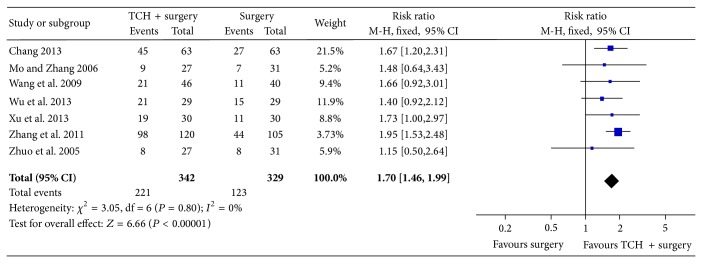
Meta-analysis of pregnancy rate at 2-year follow-up.

Seven studies reported pregnancy rate at 2-year follow-up. The results of meta-analysis using the fixed effects model indicated a significantly higher pregnancy rate in the traditional Chinese herbs combined with surgery compared to surgery control (RR = 1.70, 95% CI = 1.46 to 1.99, and *P* < 0.00001; Figure 5).

### 3.5. Secondary Outcome

No significant adverse reactions were noted.

### 3.6. Publication Bias Assessment

In this review, the use of funnel plots was limited due to the small number of studies evaluated.

## 4. Discussion

### 4.1. Summary of Evidence

To the best of our knowledge, this is the first systematic review and meta-analysis of traditional Chinese herb combined with surgery RCTs for the treatment of male varicocele infertility. In this study, 17 RCTs were identified for systematic review and meta-analysis. A total of 794 patients in the treatment groups and 747 in the control groups were evaluated and the duration of RCTs ranged from 3 months to 2 years. All of these RCTs were conducted in mainland China. Despite the poor methodological quality and small sample size, analysis of the pooled data showed a consistent superior effect of traditional Chinese herbs combined with surgery in increasing the pregnancy rate at 3-month, 6-month, and 2-year follow-ups, when compared to surgery control group.

In these RCTs, the time to take medicine could be a week after the surgery, while the dosage of Chinese medicine could be two times a day, each time 150–200 mL. The course of treatment was recommended to be 3 months.

### 4.2. Limitations and Strengths of This Review

Although our literature searches included English and Chinese databases and included searching by hand for relevant articles and registered information, we still cannot be certain that all relevant trials were found.

The quality scores of the included RCTs were generally poor. Although all studies had a randomization design, very few studies described the randomization procedure in detail. In addition, lack of information on allocation concealment and blinding of assessors indicated that important sources of bias in these RCTs could not be excluded.

Although there was no statistical heterogeneity between these trials about pregnancy rate according to Cochrane's *χ* ^2^ test, there would be unpredictable clinical heterogeneity in reality. For instance, the components of TCM, doses, and formulations used were different for each of RCTs, and different surgical methods were used to treat the male varicocele infertility.

Two recent studies have shown that the incidence of varicocele with secondary infertility is significantly greater than primary infertility [34, 35]. It was concluded that the men with secondary infertility experienced progressive deterioration of their fertility status over time, which was either directly or indirectly due to their varicocele. All the included trials in this meta-analysis neglected the difference between primary infertility and secondary infertility, which would be bias. This needs to be addressed in future clinical trials.

### 4.3. Mechanism of Traditional Chinese Herbs for Varicocele Infertility

Many scholars have recommended that surgery can improve the chances of infertile patients with varicocele fertility in different views. However the use of traditional Chinese medicine combined with surgery to improve the male varicocele infertility could be an effective method, which only exists in theory in reality. Unfortunately, there were very few RCTs on TCM for male infertility, and they included very small number of patients [36].

In the TCM theory, the kidney is the organ that stores kidney essence and plays a crucial role in reproduction. Because of the complex etiology, Chinese physicians have different views on the etiology and pathology of varicocele infertility. However, most Chinese physicians think that kidney emptying and blood stasis are basic causes of varicocele infertility and TCM therapy can supplement the kidney essence and promote the blood circulation to remove blood stasis and thereby cure the varicocele infertility [37–40].

Substantial pharmacological researches have been performed to investigate the chemical constituents of a variety of TCMs. Many of the TCMs have strong antioxidant activity and function like gonadotropins, which can increase the production of testosterone and improve the sperm motility. Wang et al. [41] found that Wu-Zi-Yan-Zong-Wan can have protective effect on oxidative damage of mitochondrial deoxyribonucleic acid in aged men. Chinese herbal medicine, Fu Pen Zi (palm-leaf raspberry fruit), can increase testosterone levels of rats but reduce the estradiol levels [42]. Ba Ji Tian (radix morindae officinalis) has been shown to increase the production of testosterone and has protective effects against hydrogen peroxide-induced oxidative stress [43]. Tu Si Zi (Semen Cuscutae) can markedly improve the sperm motility [44]. Many Chinese herbal medicines possess strong antioxidant activity, which play a role in stabilizing the sperm membrane and reduce the lipid peroxidation of the sperm plasma membrane that may result in sperm dysfunction and cell death [45]. There are some other Chinese herbal medicines for male infertility, which may increase the trace elements [46, 47].

## 5. Conclusion

It is not possible to recommend traditional Chinese herbs as a proven treatment for male varicocele infertility on the basis of published evidences. However, this evidence is derived from inadequate clinical trials. Studies on male varicocele infertility have frequently demonstrated marked improvements in the placebo group, and thus well-controlled studies are required to demonstrate true effects.

## Figures and Tables

**Table 1:** Description of the characteristics of trials included in the meta-analysis.

Study	Sample size	Age	Duration	Duration of treatment	Treatment	
	T/C	T/C	T/C (years)	M	Experimental group	Control group
Cao 2006 [27]	27/27	28.63/28.48	2.89/2.85	3	Yishentongluo granules + surgery	High ligation of the spermatic vein
Chang 2013 [29]	63/63	27.9/26.8		3	Guizhifuling capsules + surgery	High ligation of the spermatic vein
Chen et al. 2012 [31]	50/50			6	Wuziyanzong pills + Shengjing capsules + surgery	High ligation of the spermatic vein
Chen and Li 2011 [25]	40/38	27		3–6	Bushenquyushengjin decoction + Danshen injection + surgery	High ligation of the spermatic vein
Cui 1998 [20]	37/37	27.98/29.52	3.38/1.08	3	Tongjinling decoction + surgery	High devascularization
He et al. 2012 [26]	40/40			6	Shengjingzhuyu decoction + surgery	High ligation of the spermatic vein
Hu 2009 [32]	60/30			3	Modified Wuziyanzong pills + surgery	High ligation of the spermatic vein
Li 2008 [21]	20/20	31.56/30.13	3.86/4.19	3	Zhuangjinzhuyu decoction + surgery	Modified Palomo high ligation of the spermatic vein
Mo and Zhang 2006 [22]	27/31			3	Traditional Chinese herbal decoction + surgery	Modified Palomo high ligation of the spermatic vein
Sun et al. 2012 [30]	57/53	31.3		4	Zhuyutongmai capsules + surgery	High ligation of the spermatic vein
Tang 2009 [28]	45/43			3	Yiqishengjin granules + surgery	Laparoscopic high ligation of the spermatic vein
Wang and Ma 2012 [19]	76/86	26/27		3	Spermatogenic tablets + surgery	Retroperitoneal high ligation of the spermatic vein
Wang et al. 2009 [23]	46/40			3	Shengjingzhuyu decoction + surgery	Retroperitoneal laparoscogation operation
Wu et al. 2013 [33]	29/29	27.5/28.53		3	Wuziyanzong pills + surgery	High ligation of the spermatic vein
Xu et al. 2013 [17]	30/30	28		3	Tongpizanyu decoction + surgery	Inguinal high ligation of the spermatic vein
Zhang et al. 2011 [18]	120/105	28.6		2	Bushenzhuangyangyuzi pills + surgery	Retroperitoneal high ligation of the spermatic vein
Zhuo et al. 2005 [24]	27/31			3	Traditional Chinese herbal decoction + surgery	Modified Palomo high ligation of the spermatic vein
